# Enhanced RAGE Expression and Excess Reactive-Oxygen Species Production Mediates Rho Kinase-Dependent Detrusor Overactivity After Methylglyoxal Exposure

**DOI:** 10.3389/fphys.2022.860342

**Published:** 2022-03-28

**Authors:** Akila L. Oliveira, Matheus L. Medeiros, Mariana G. de Oliveira, Caio Jordão Teixeira, Fabíola Z. Mónica, Edson Antunes

**Affiliations:** ^1^ Department of Pharmacology, University of Campinas (UNICAMP), Campinas, Brazil; ^2^ Department of Physiology and Biophysics, Institute of Biomedical Science, University of Sao Paulo, Sao Paulo, Brazil

**Keywords:** advanced glycation end products, rage, urothelium, Y27632, superoxide dismutase, L-type Ca^2+^ channels

## Abstract

Methylglyoxal (MGO) is a highly reactive dicarbonyl compound implicated in diabetes-associated diseases. In vascular tissues, MGO induces the formation of advanced glycation end products (AGEs) that bounds its receptor RAGE, initiating the downstream tissue injury. Outside the cardiovascular system, MGO intake produces mouse voiding dysfunction and bladder overactivity. We have sought that MGO-induced bladder overactivity is due to activation of AGE-RAGE-reactive-oxygen species (ROS) signaling cascade, leading to Rho kinase activation. Therefore, female mice received 0.5% MGO orally for 12 weeks, after which *in vitro* bladder contractions were evaluated in the presence or not of superoxide dismutase (PEG-SOD) or the Rho kinase inhibitor Y27632. Treatment with MGO significantly elevated the serum levels of MGO and fluorescent AGEs, as well as the RAGE immunostaining in the urothelium, detrusor, and vascular endothelium. RAGE mRNA expression in the bladder was also higher in the MGO group. Methylglyoxal significantly increased the ROS production in both urothelium and detrusor smooth muscle, with the increases in detrusor markedly higher than urothelium. The bladder activity of superoxide dismutase (SOD) was significantly reduced in the MGO group. Gene expressions of L-type Ca^2+^ channels, RhoA, ROCK-1, and ROCK-2 in bladder tissues were significantly elevated in the MGO group. Increased bladder contractions to electrical-field stimulation, carbachol α,β-methylene ATP, and extracellular Ca^2+^ were observed after MGO exposure, which was significantly reduced by prior incubation with either PEG-SOD or Y27632. Overall, our data indicate serum MGO accumulation elevates the AGEs levels and activates the RAGE-ROS signaling leading to Rho kinase-induced muscle sensitization, ultimately leading to detrusor overactivity.

## Introduction

Methylglyoxal (MGO) is a highly reactive dicarbonyl compound generated during glycolysis in the presence of high glucose levels (Rabbani and Thornalley, 2015). High levels of MGO are found in the plasma of patients with diabetes mellitus (Kilhovd et al., 2003; Han et al., 2009), and this high glucose-mediated increase of MGO production is believed to account for the cell damage in diabetes-associated pathological conditions (Liu et al., 2012). MGO modifies lysine, arginine, and cysteine residues in peptides or proteins to yield irreversible advanced glycation end products (AGEs), leading to cross-linking and denaturation of proteins (Liu et al., 2012). AGEs bound its receptor RAGE, which is a member of the immunoglobulin superfamily of cell surface receptors that can recognize endogenous ligands (Kim et al., 2021). Upon activation, RAGE triggers multiple intracellular signaling pathways, including the activation of NADPH oxidase, producing excess reactive-oxygen species (ROS) levels (Nowotny et al., 2015; Rabbani and Thornalley, 2015; Mey and Haus, 2018).

RhoA/Rho-kinase activation by different GPCR agonist plays a critical role in the regulation of actin cytoskeleton dynamics and generation of actin-myosin contractility in vascular and non-vascular smooth muscles (Peters et al., 2006; Shimokawa et al., 2016). Rho kinase is a serine/threonine kinase with a molecular weight of 160 kDa, and comprises two isoforms, ROCK1 and ROCK2 (Nunes and Webb, 2020). The activity of RhoA is controlled by the guanine nucleotide exchange factors (GEFs) that catalyze the exchange of the inactive Rho-GDP for the active Rho-GTP state, which interacts with the downstream effector Rho kinase, ultimately leading to Ca^2+^ sensitization and contraction (Nunes and Webb, 2020). The smooth muscle contraction involves a balance between myosin light chain kinase (MLCK) and myosin light chain phosphatase (MLCP), where the MLC phosphorylation enhances the contractile mechanism. MLCP enzyme consists of three subunits, namely, the catalytic subunit PP1cδ, the regulatory subunit MYPT1, and a 20 kDa subunit. MYPT1 modulates Ca^2+^-dependent phosphorylation of MLC by MLCK. Activation of Rho-kinase regulates the phosphorylation level of MLC by directly phosphorylating this enzyme and by inactivating MLCP through phosphorylation of MYPT1 at Thr697 and Thr855, thus enhancing the MLC phosphorylation (MacDonald and Walsh, 2018). In addition, the RhoA/Rho kinase pathway is one of the major intracellular pathways that enhance the expressions of molecules for oxidative stress (Hobbs et al., 2014; Strassheim et al., 2019). Previous studies showed that reactive-oxygen species (ROS) enhances the Rho-GTPase activity (Heo and Campbell, 2005; Aghajanian et al., 2009). In pulmonary vascular smooth muscle under chronic hypoxia, ROS generation was shown to mediate the Ca^2+^ sensitization and vasoconstriction through upregulation of RhoA/ROCK signaling (MacKay et al., 2017; Yan et al., 2020). The impairment of corpus cavernosum relaxations diabetic mice were associated with upregulation of the RhoA/Rho-kinase signaling pathway and increased oxidative stress (Li et al., 2012; Priviero et al., 2016). In isolated cells, Rho kinase activation also increase the ROS production (Lu et al., 2013; Fujimoto et al., 2017; Cap et al., 2020).

Metabolic diseases are clinically characterized by elevated blood glucose levels that are associated with complications such as kidney and heart disease, and microvascular and macrovascular alterations. Outside the cardiovascular system, metabolic factors play important roles in the development of overactive bladder syndrome (OAB), urinary incontinence, and other lower urinary tract symptoms (LUTS) (Lai et al., 2019; Wittig et al., 2019), which may be due to alterations at the level of the detrusor smooth muscle, urothelium and neuronal innervation (Daneshgari et al., 2017). In animal models of diabetes, micturition dysfunction is characterized by increases in void number, volume, and capacity, despite conflicting data have been obtained depending on the model used (Gasbarro et al., 2010; Leiria et al., 2011; Leiria et al., 2012; Wu et al., 2016; Ellenbroek et al., 2018; Yang et al., 2019; Kim et al., 2020). More recently, we have reported a model of bladder dysfunction induced by 4- and 12-weeks intake of MGO to healthy male mice (de Oliveira et al., 2020; Oliveira et al., 2021), which differs from the classical diabetic animals in that MGO is not generated from the endogenous glucose metabolism and therefore does not affect the glucose levels and insulin sensitivity (Medeiros et al., 2020). Urodynamic evaluation of MGO-treated mice revealed significant increases of micturition frequency and the number of non-voiding contractions (NVCs), along with enhanced *in vitro* bladder contractility to muscarinic and P2X1 receptor activation. We tested here the hypothesis that MGO-induced bladder overactivity is a consequence of the activation of the AGE-RAGE-ROS axis, ultimately leading to upregulation of the Rho kinase system in detrusor smooth muscle. Therefore, in intact or mucosa-denuded mouse bladder strips, we carried out molecular and biochemical assays for RAGE, ROS, L-type Ca^2+^ channels, and Rho kinase expressions and evaluated the *in vitro* contractile responses in the presence of superoxide dismutase (SOD) and the Rho kinase inhibitor Y27632.

## Materials and Methods

### Animals and Treatment With MGO

Five-week-old female C57BL/6 mice weighing 18 ± 0.30 g at the beginning of the study were housed in cages (three mice per cage) made of polypropylene with dimensions 30 × 20 × 13 cm) located in a ventilated cage shelters with a constant humidity of 55 ± 5% and temperature of 24 ± 1°C under a 12 h light-dark cycle. The animals were acclimated for 7 days before starting the treatments. Animals received standard food *ad libitum* and filtered water. Animal procedures and experimental protocols were approved by Ethics Committee in Animal Use, State University of Campinas (CEUA-UNICAMP; Protocol No. 5443-1/2019). The animals were randomly assigned using a number randomization calculator (available at https://www.graphpad.com). Animals received 0.5% MGO (Sigma Aldrich, Missouri, United States) in the drinking water for 12 weeks, whereas control mice received tap water alone. The doses and route of administration of MGO used were chosen according to a previous study in mice (Medeiros et al., 2020). Body weights, water consumption, and food consumption were assessed weekly in all groups. Animal studies are reported in compliance with the ARRIVE guidelines.

### Measurements of Levels of MGO, Fluorescent AGEs and Glucose

Peripheral blood (0.5 ml) was collected by the intracardiac puncture. Blood was centrifuged at 5,000 × g for 10 min at 4°C and serum was transferred to microcentrifuge tubes. To measure MGO levels in serum, the samples were deproteinized using Deproteinizing Sample Preparation Kit - TCA (Abcam ab204708) according to the manufacturer’s instructions. Serum levels of MGO were measured using ELISA competitive kit for OxiSelect™ Methylglyoxal (Catalog No. STA-811, Cell Biolabs, San Diego, CA, United States). F-AGEs in serum were measured diluting the samples 1:2 in phosphate-buffered saline (PBS, pH 7.4), and transferred to black 96-well plates, 200 µl total per well. Fluorescence spectra were recorded in duplicate on a fluorometer (SynergyTM H1 Hybrid Reader, Biotek, United States). The excitation and emission wavelengths were 360 and 447 nm, respectively, and the PBS solution was used as blank. Individual concentrations were normalized by blank fluorescence. The fluorescence intensity was expressed in arbitrary units (a.u.). To assess glucose concentration, animals fasted for 8 h, blood from the tail vein was collected and glucose was measured using ACCUCHECK Blood Glucose (Monitoring System^®^, Roche Diagnostics, Indianapolis, United States).

### Measurements of Superoxide Dismutase Activity in Bladder Tissue

For the measurement of SOD activity, bladder tissue was homogenized following the manufacturer’s protocol, and bladder homogenate (supernatant) was used for measurement of enzymatic SOD assay kit (Cayman Chemical, Catalog No 706002, Ann Arbor, MI, United States).

### Bladder Histology

The animals were weighed and anesthetized with isoflurane in a concentration greater than 5% and cervical dislocation was performed to confirm the euthanasia. The bladder was removed and weighed wet for determination of the relative bladder weight (bladder to body ratio). The bladder was fixed with 10% phosphate-buffered formalin for 24 h, dehydrated in ethanol, and embedded in paraffin. Tissues were sliced (5-μm sections) on a microtome (Leica, Wetzlar, Germany), dewaxed in xylene, rehydrated in gradient alcohol, and stained with Haematoxylin & Eosin (H&E) and Masson trichrome for light microscopy examination. Digital images were obtained with a microscope Eclipse 80i (Nikon, Tokyo, Japan) equipped with a digital camera (DS-U3, Nikon). The thickness of urothelium and detrusor smooth muscle, and the collagen content were evaluated using the Fiji version of the ImageJ Software (Version 1.46r), according to a previous study (Oliveira et al., 2021).

### Immunohistochemistry for RAGE

Bladder immunoperoxidase reactions were processed based on previous studies (Medeiros et al., 2021). Briefly, bladder samples were removed, immersed in 10% formalin fixative solution for 48 h, and embedded in paraffin. Five-micron sections were mounted onto aminopropyltriethoxysilane-coated glass slides. Sections were deparaffinized, rehydrated, and washed with 0.05 M Tris buffer solution (TBS) at pH 7.4. Subsequently, for antigen retrieval, sections were treated with 0.01 M Tris-EDTA buffer containing 0.05% Tween-20 (pH 9.0) for 24 min at 98°C. Endogenous peroxidase activity was inhibited with 0.3% hydrogen peroxide (H_2_O_2_) solution. For blocking the non-specific sites, a 5% bovine serum albumin (BSA) solution containing 0.1% Tween-20 for 60 min was used. Sections were incubated with mouse monoclonal anti-RAGE primary antibody (1:70; Cat. No. ab54741, Abcam, Cambridge, United Kingdom) diluted in TBS containing 3% BSA overnight at 4°C. Subsequently, sections were washed, and incubated with biotinylated goat anti-mouse IgG, avidin and biotinylated HRP (1:20; Cat. No. EXTRA2, Sigma Aldrich, St Louis, MO, United States) following all the manufacturer’s instructions. For detection of the immunostained area with RAGE, a 3.3′ diaminobenzidine solution (DAB; cat. no. D4293, Sigma Aldrich) was employed. As a negative control, a section was used in parallel to primary antibody omission. As a positive control, sections of mouse lungs were used (Medeiros et al., 2021). All slides were counterstained with hematoxylin and mounted for observation by microscopy. Representative images were acquired using a light microscope Leica DM 5000B (Leica Microsystems Inc., Buffalo Grove, IL, United States) equipped with a digital camera under a ×40 objective.

### Measurement of ROS Levels in Bladder Tissues

The oxidative fluorescent dye dihydroethidium (DHE) was used to evaluate *in situ* ROS generation. The bladders were embedded in a freezing medium and transverse sections (12-μm) were obtained on a cryostat, collected in glass slides, equilibrated for 10 min in Hank’s solution (1.6 mM CaCl_2_, 1.0 mM MgSO_4_, 145.0 mM NaCl, 5.0 KCl, 0.5 mM NaH_2_PO_4_, 10.0 mM Glucose, 10.0 HEPES, pH 7.4). Fresh Hank’s DHE solution (2 μM) was applied to each tissue section, and the slides were incubated in a light-protected humidified chamber at 37°C for 30 min. Images were obtained with a microscope (Eclipse 80i, Nikon, Tokyo, Japan) equipped for epifluorescence (excitation at 488 nm; emission at 610 nm) and a digital camera (DS-U3, Nikon). Fluorescence was detected with a 585 nm long-pass filter. The number of nuclei labeled with ethidium bromide was automatically counted in separated detrusor smooth muscle and urothelium using ImageJ software (NIH, Maryland, United States), and expressed as labeled nuclei per square millimeter.

### Quantitative Real-Time RT-PCR (qPCR) for RAGE, L-type Ca^2+^ Channels (Cacn1), RhoA, ROCK1 and ROCK2

Total RNA was extracted from freshly dissected bladders using TRIzol^®^ reagent (Invitrogen, MS, United States), according to the manufacturer’s protocol. Dnase treated RNA samples were then transcribed with High-Capacity Reverse Transcription Kit^®^ (Applied Biosystems, CA, United States). cDNA samples concentrations were quantified using a spectrophotometer (Nanodrop Lite^®^, Thermo Scientific, Massachusetts, United States). Synthetic oligonucleotide primers (Table 1) were obtained from Integrated DNA Technologies (Iowa, United States), Exxtend Oligo Solutions (Brazil) and Qiagen Quantitec Primeras Assays (Germany). The reactions were performed with 10 ng cDNA, 6 µl SYBR Green Master Mix^®^ (Life Technologies, CA, United States), and the optimal primer concentration in a total volume of 12 µl. Real-time PCR was performed in the equipment StepOne-Plus^®^ Real Time PCR System (Applied Biosystems). The reaction program was 95°C for 10 min, followed by 40 cycles of 95°C for 15 s then 60°C for 1 min. At the end of a normal amplification, a degradation time was added, during which the temperature increased gradually from 60 to 95°C. Threshold cycle (Ct) was defined as the point at which the fluorescence rises appreciably above the background fluorescence. To determine the specificity of the amplification, the melting curve analysis of the PCR products was performed to ensure that only one fragment was amplified. To determine the specificity of the amplification, the melting curve analysis of the PCR products was performed to ensure that only one fragment was amplified. The 2^-ΔΔCt^ method was utilized to analyze the results, which were expressed by the difference between Ct values of chosen genes and the housekeeping gene β–actin. The signal strength for β–actin did not differ between groups (Ct: 19.06 ± 0.33 and 18.95 ± 0.24 for control and MGO respectively).

**TABLE 1:** Primer sequences used for real-time PCR amplifications.

Gene	Forward	Reverse
RAGE (IDT Integrated DNA Tecnhologies)	5′-CTG​AAC​TCA​CAG​CCA​GTG​TCC​C-3′	5′-CCC​TGA​CTC​GGA​GTT-3′
Cacn1 (Exxtend Oligo Solutions)	5′-ACC​CTC​CTC​CGT​CGA​ATT​C-3′	5′-GTG​TGC​CAT​CGC​TGT​TCA​GA-3′
RHOA (Exxtend Oligo Solutions)	5′-CCT​TCG​GAA​TGA​CGA​GCA​C-3′	5′-AGA​TGA​GGC​ACC​CAG​ACT​TTT-3′
ROCK1 (Exxtend Oligo Solutions)	5′-ACC​CAC​CAT​CTG​GCT​TTG​TC-3′	5′-CGG​TTT​ATC​AGG​TAG​CAT​CCC-3′
ROCK 2 (Exxtend Oligo Solutions)	5′-GAT​GGT​TGT​CAT​TGC​CTG​TGC-3′	5′-TGC​TCT​TTA​TCT​TGT​TCG​CTG​T-3′
ACTB (Qiagen Quantitec Primers Assays)	QT00095242	NM_007,393

### Bladder Preparation and Organ Bath Set-up

The animals were weighed and anesthetized with isoflurane in a concentration greater than 5% and cervical dislocation was performed to confirm the euthanasia. The bladder was removed and weighed wet for determination of the relative bladder weight (bladder to body ratio), after which it was carefully divided into 2 strips. In one bladder strip, the urothelium with the lamina propria was carefully removed using fine-tip forceps whereas the other strip remained with the mucosa intact. Strips were mounted in 10 ml organ baths containing Krebs−Henseleit solution (117 mM NaCl, 4.7 mM KCl, 2.5 mM CaCl_2_, 1.2 mM MgSO_4_, 1.2 mM KH_2_PO_4_, 25 mM NaHCO_3_ and 11 mM Glucose, pH 7.4) continuously bubbled with a mixture of 95% O_2_ and 5% CO_2_. Tissues were allowed to equilibrate for 45 min under resting tension and then adjusted for 5 mN. Changes in isometric force were recorded using a PowerLab system (ADInstruments Inc., Sydney, AU).

### Bladder Contractions Induced by Electrical-Field Stimulation

EFS was applied to intact and mucosa-denuded bladder strips placed between two platinum ring electrodes connected to a stimulator (Grass Technologies, Rodhe Island, United States). EFS was conducted at 80 V, 1 ms pulse width, and trains of stimuli lasting 10 s at varying frequencies (1–32 Hz) with 2 min intervals between stimulations. In a separate set of experiments of mucosa-denuded bladders, strips were preincubated (30 min) with either the Rho-kinase inhibitor Y27632 (1 µM) (Catalog No Y0503, Sigma-Aldrich, United States) or superoxide dismutase-polyethylene glycol (PEG-SOD; 87 IU/ml; Catalog No S9549, Sigma-Aldrich, United States) prior to application of EFS. The contractile responses were expressed as mN/mg.

### Concentration-Response Curves to Carbachol and α,β-Methylene ATP

Cumulative concentration-response curves to the muscarinic receptor agonist carbachol (1 nM–100 μM; Sigma Aldrich, Missouri, United States) were constructed in both intact and mucosa-denuded preparations. In the mucosa-denuded strips, Y27632 (1 µM) or PEG-SOD (87 IU/ml) was preincubated (30 min) prior to construction of concentration-response curves to carbachol. Non-linear regression analysis to determine the potency (pEC_50_) of carbachol was carried out using GraphPad Prism (GraphPad Software, Inc., California, United States) with the constraint that F = 0. Concentration-response data were fitted to a log dose-response function with a variable slope in the form: E = Emax/([1 + (10c/10x) n] + F), where E is the effect of above basal, Emax is the maximum response produced by agonists; c is the logarithm of the pEC_50_, the concentration of drug that produces a half maximal response; x is the logarithm of the concentration of the drug; the exponential term, *n*, is a curve-fitting parameter that defines the slope of the concentration–response line, and F is the response observed in the absence of added drug.

In separate bladder strips, non-cumulative curves to the P2X1 purinergic agonist α,β-methylene ATP (1, 3 and 10 μM; Sigma Aldrich, Missouri, United States) were constructed in intact and mucosa-denuded bladder strips using 20-min intervals between concentrations to avoid tachyphylaxis. The contractile responses were expressed as mN per milligram of wet tissue (mN/mg). To verify the viability of the preparations, 80 mM KCl solution was added to the baths at the end of equilibration time.

### Pharmacological Evaluation of Bladder Contraction to Extracellular Ca^2+^ Influx

Cumulative concentration-response curves to extracellular CaCl_2_ (10 μM–100 mM) in intact and mucosa-denuded bladder strips were constructed according to a previous study (Leiria et al., 2011). Briefly, tissues were equilibrated for 45 min in 10 mL-organ baths containing Krebs-Henseleit solution, after which the bath solution was removed and replaced by a Ca^2+^-free Krebs solution containing EGTA (1 mM) to sequester Ca^2+^ ions. The preparations were contracted with KCl (80 mM) followed by contractions with carbachol (10 µM) to deplete intracellular Ca^2+^. Next, preparations were incubated with cyclopiazonic acid (CPA; 10 µM) for 30 min to block sarcoplasmic reticulum Ca^2+^ stores. Concentration-responses curves to CaCl_2_ were then constructed. Mucosa-denuded bladder strips were preincubated (30 min) with either Y27632 (1 µM) or PEG-SOD (87 IU/ml) before construction of concentration-response curves to CaCl_2_.

### Statistical Analysis

Data were expressed as the mean ± standard error of the mean (SEM) of 23 mice (bladder measures) or 5 to 8 (other parameters) per group. The GraphPad Prism Version 6 Software (GraphPad Software Inc.) was used for all statistical analysis. Statistical difference between groups was determined by ANOVA and Tukey’s post-test. Student’s unpaired *t*-test was used when appropriate. *p* < 0.05 was accepted as significant.

## Results

### Levels of Methylglyoxal, F-AGEs and Glucose

**FIGURE 1:**
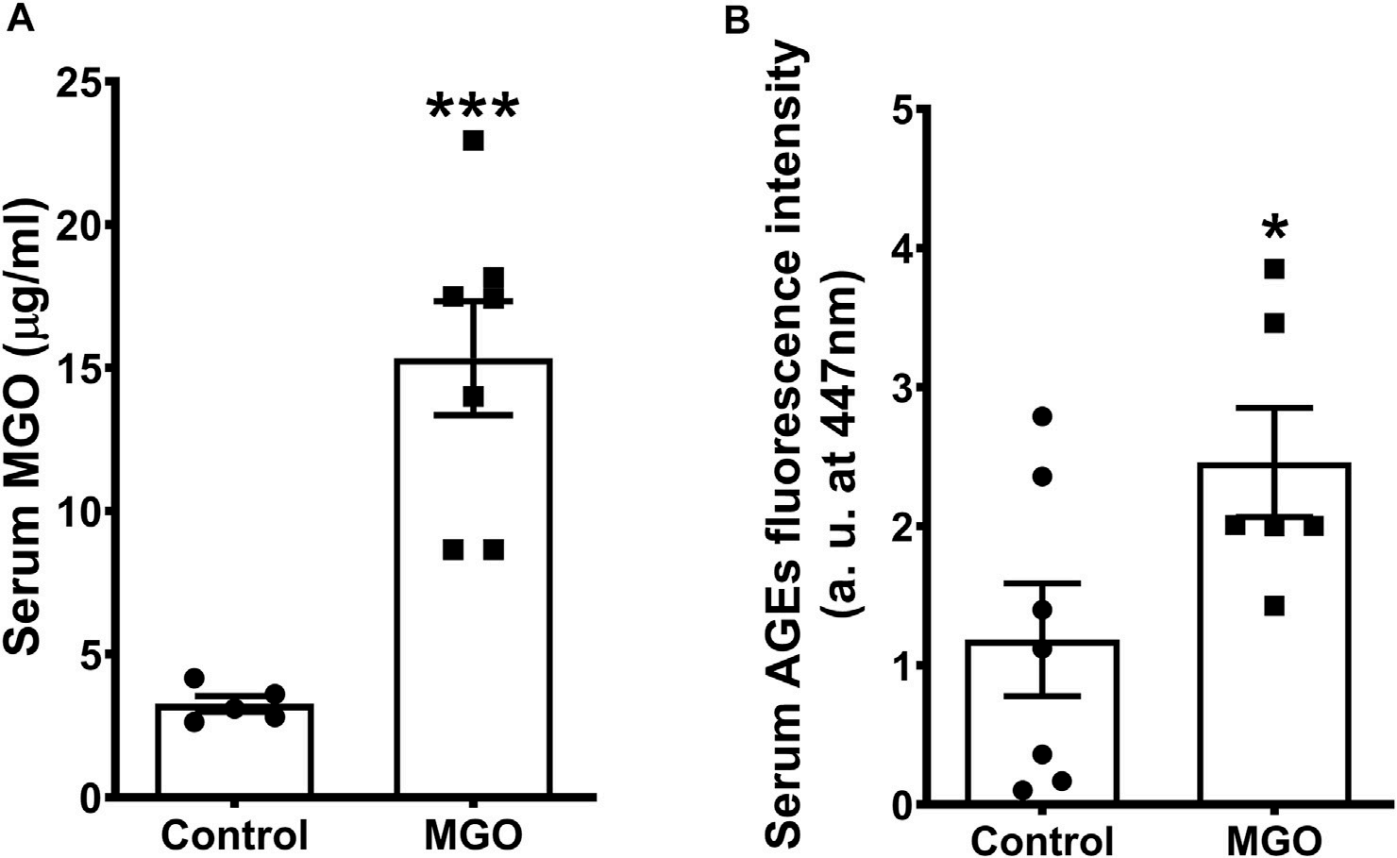
Serum levels of methylglyoxal (MGO) **(A)** and fluorescent advanced glycation end products (F-AGEs **(B)** in mice receiving or not 0.5% MGO for 12 weeks in the drinking water. Data are expressed as mean ± SEM (*n* = 5−7). *p < 0.05, ***p < 0.001 compared with control group (unpaired *t*-test).

The serum MGO concentration after 12-weeks oral dose of 0.5% MGO was 4.8-fold higher than control mice (*p* < 0.01; Figure 1A). Serum levels of F-AGEs were also significantly increased in MGO compared with control group (*p* < 0.05; Figure 1B). The levels of fasting glucose (86.8 ± 5.43 mg/dl), water consumption (2.45 ± 0.22 ml/day) and food intake (3.4 ± 0.11 g/day) in MGO group did not differ significantly from control group (89.7 ± 4.63 mg/dl, 2.61 ± 0.09 ml/day, and 3.2 ± 0.11 g/day, respectively; n = 7).

### Bladder Measures and Histology

**FIGURE 2:**
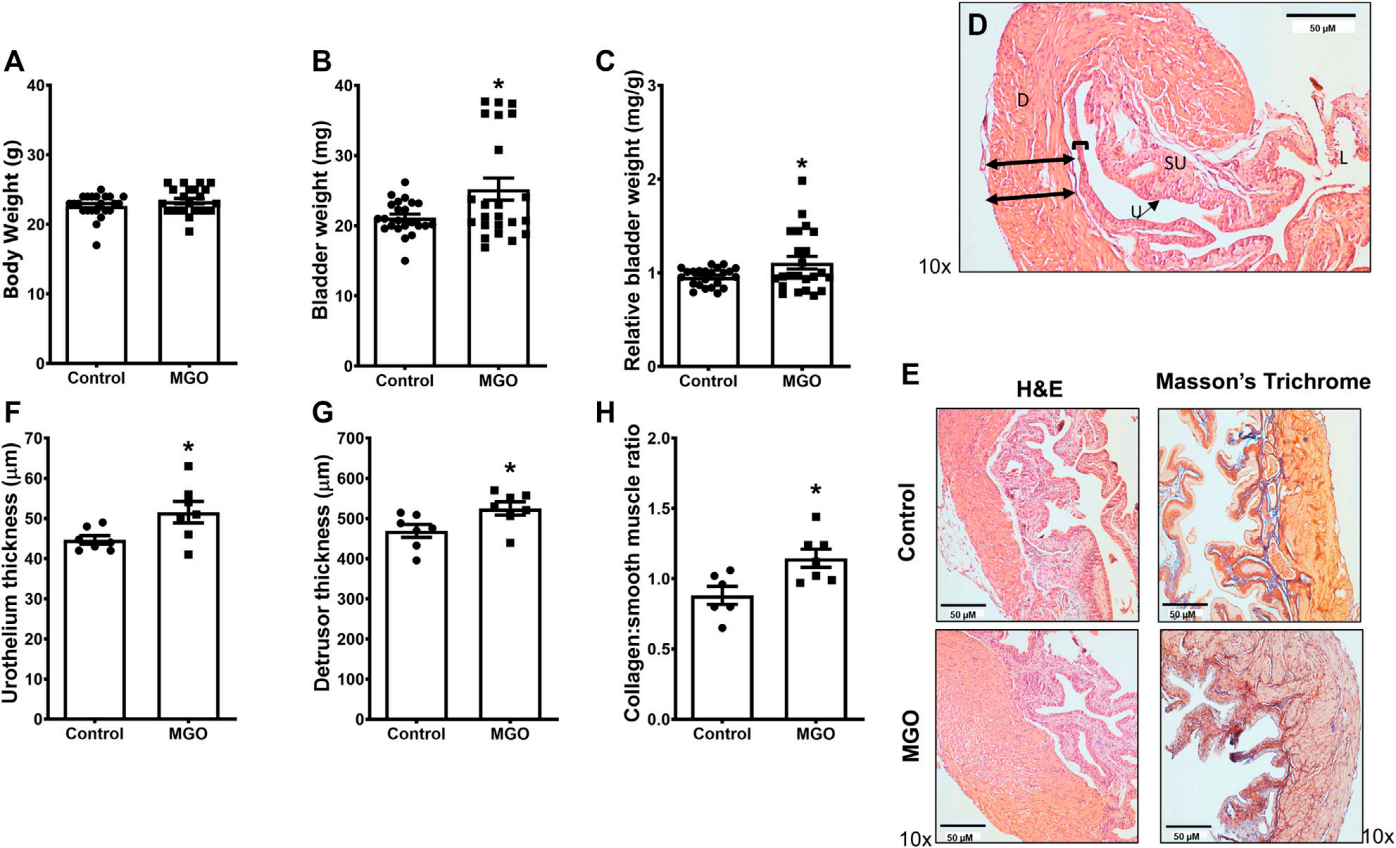
Bladder measures and histological evaluation of methylglyoxal (MGO)-treated mice in comparison with control animals. Panels **(A−C)** show body weight, bladder weight and relative bladder weight, respectively, in control and MGO groups. Panel **(D)** shows a histological image of a bladder from control mice indicating the urothelium and detrusor smooth muscle with double-black arrows indicating the limits of quantification in detrusor and brackets indicating limits of quantification in urothelium. Panel **(E)** shows representative histological images of bladders of control and MGO groups using cross-section hematoxylin and eosin (HE) and Masson’s trichrome staining. Panels **(F−H)** show quantifications of urothelium thickness, detrusor thickness and collagen content, respectively. Data are expressed as mean ± SEM (*n* = 23 for body weights; *n* = 7 for urothelium thickness, detrusor thickness and collagen content). *p < 0.05 compared with control group (unpaired *t*-test). **D**, detrusor smooth muscle; **SU,** suburothelium, **U**, urothelium; **L,** lumen. Black bars in panels **D** and **E** represent a scale of 50 µm (×10 objective).

The body weight did not significantly differ between control and MGO groups (Figure 2A), but the bladder weight and relative bladder weight were increased by about of 20% (*p* < 0.05) in MGO-treated mice (Figures 2B,C). Histological analysis of bladders by hematoxylin and eosin (HE) and Masson’s trichrome stains (Figures 2D,E) revealed that MGO exposure caused small (despite significant at *p* < 0.05) increases of the thickness of both urothelium and detrusor smooth muscle layers as well as of the collagen content (Figures 2F–H).

### Enhanced RAGE Expression in Bladders by MGO Exposure

**FIGURE 3:**
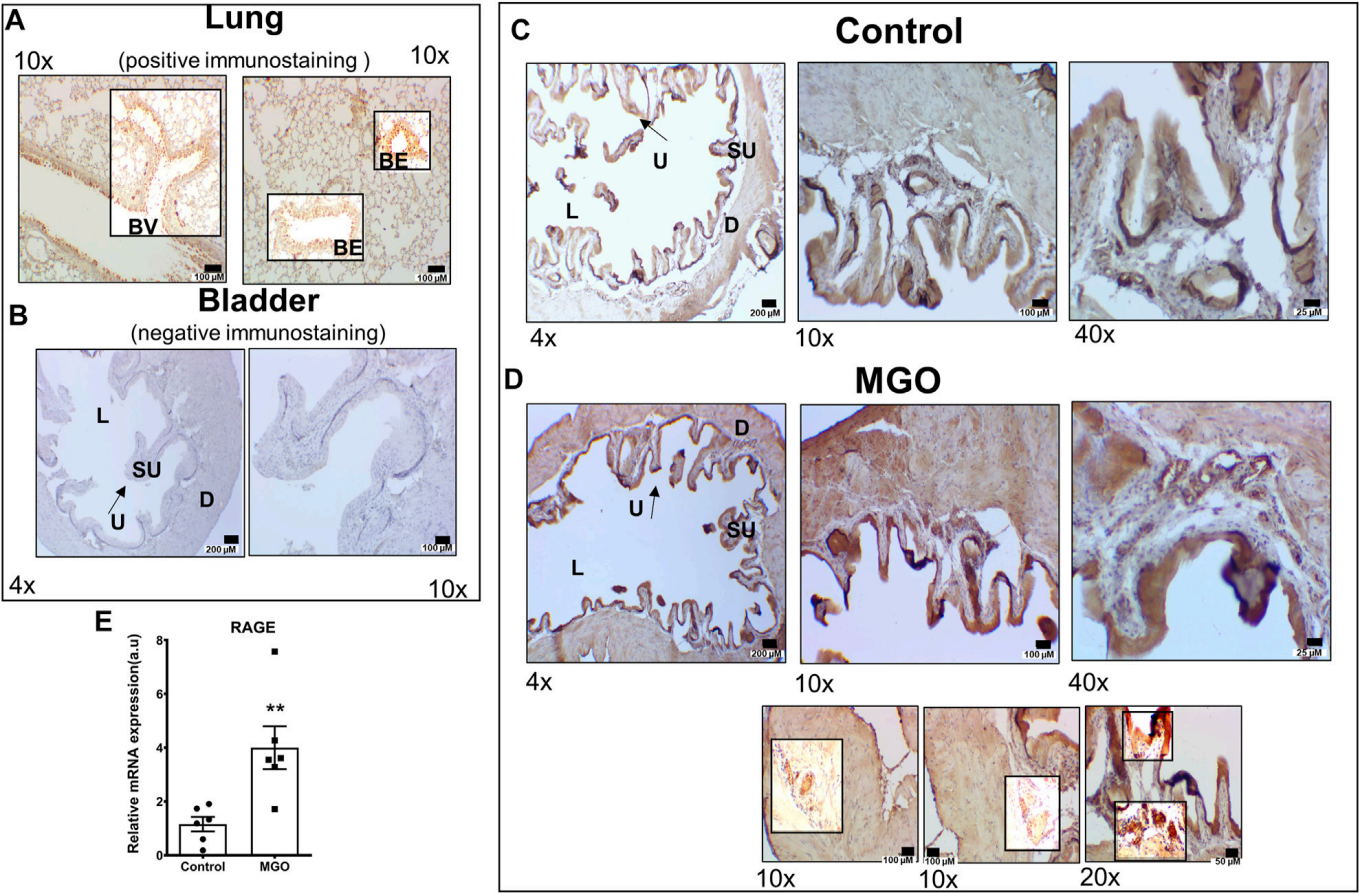
RAGE Immunostaining and mRNA expression in control and MGO-treated mice. Panel **(A)** shows RAGE immunohistochemistry in lungs of healthy mice (brown staining) as positive control, detailing the bronchial epithelium (BE) and blood vessels (BV). Panel **(B)** shows negative control (omission of antibody) in mouse bladder, detailing the urothelium (U), suburothelium (SU), lumen (L) and detrusor smooth muscle (D). Panel **(C,D)** show, respectively, RAGE immunoreactivity of bladder images of control groups MGO groups. The three sub-panels in panel **(D)** (MGO group) detail the immunostaining for RAGE in vessels supplying the detrusor and urothelium, as well as in the superficial layer of urothelium and vessels in sub-urothelium. In the bladder of control mice, RAGE immunoreactivity was observed in urothelium only, whereas in MGO group, a strong RAGE immunoreactivity was observed in urothelium, detrusor and vascular endothelium. Panel E shows RAGE mRNA expressions in bladder tissues with data expressed as mean ± SEM of arbitrary units (a. u.; **p < 0.01 compared with control, n = 6). Black bars in panels A to D represent scales of 200 μm (×4 objective), 100 μm (×10 objective), 50 μm (×20 objective) and 25 μm (×40 objective).

Immunohistochemistry for RAGE was evaluated in lungs of healthy mice (as positive control) and bladders of untreated and MGO-exposed mice. Mouse lungs clearly immunostained for RAGE as detected mainly in peribronchiolar and perivascular regions of the lung (Figure 3A). In bladders of control group, RAGE immunoreactivity was observed in urothelium only (Figure 3C) whereas in MGO group an intense RAGE immunoreactivity was observed in both urothelium and detrusor, as well as in the vascular endothelium supplying the detrusor and lamina propria (Figure 3D). Analysis of mRNA expressions of RAGE in bladder tissues revealed a significantly higher expression in MGO in comparison with control group (*p* < 0.01; Figure 3E).

### Enhanced ROS Levels and Decreased SOD Activity in Bladders by MGO Exposure

**FIGURE 4:**
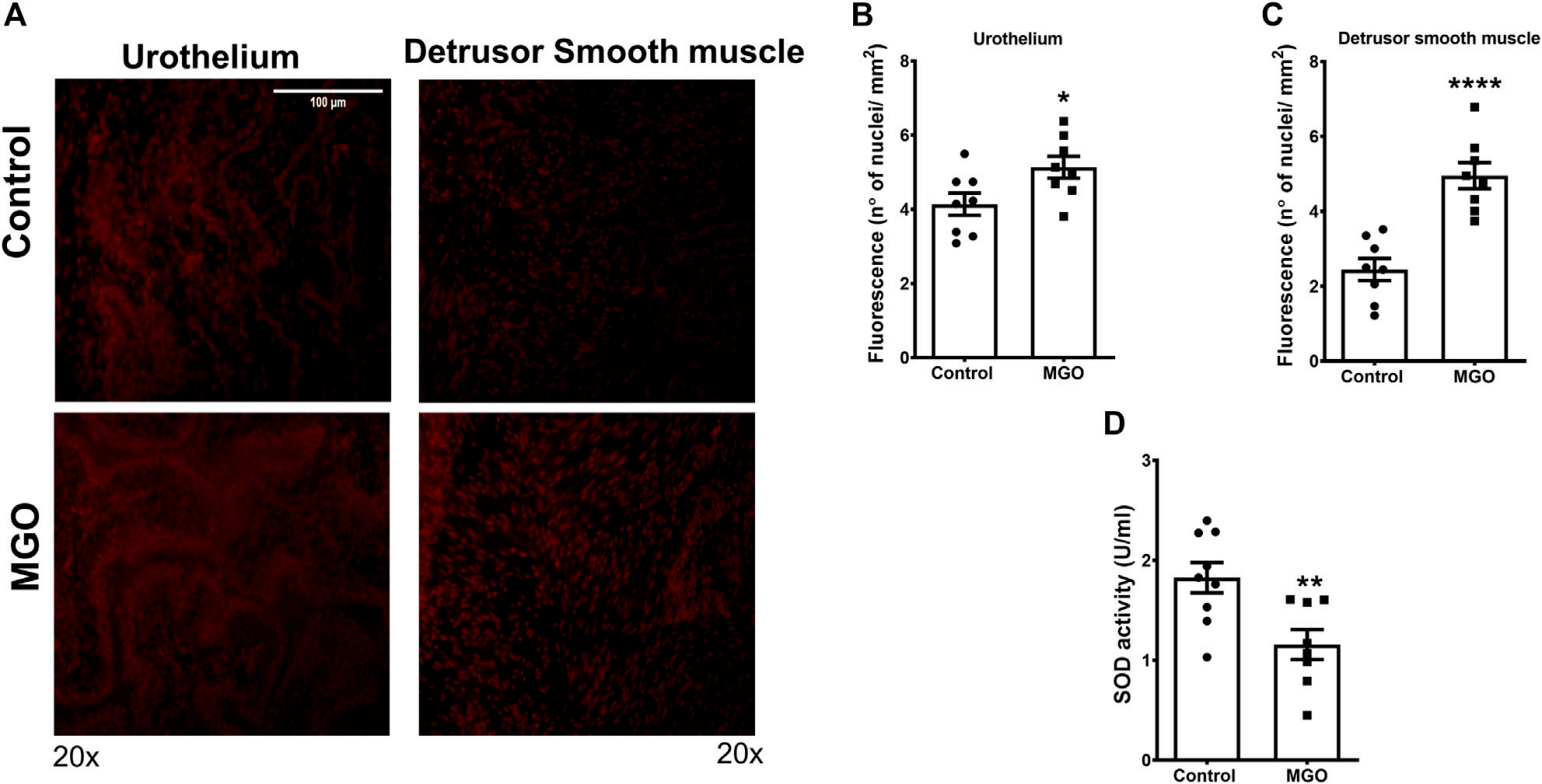
Methylglyoxal (MGO) treatment increases the reactive-oxygen species (ROS) levels in urothelium and detrusor smooth muscle and decreases SOD activity in bladders. Panel **(A)** shows representative images of ROS measurement through dye dihydroethidium-induced fluorescence in urothelium and detrusor smooth muscle in control and MGO groups (20×). Panels **(B,C)** show quantitative ROS levels in urothelium and detrusor smooth muscle, respectively. Panel **(D)** shows superoxide dismutase (SOD) activity (*n* = 8). Data are expressed as mean ± SEM. *p < 0.05, **p < 0.01, ****p < 0.0001 compared with control group. White bar in panel A represents a scale of 100 um (×20 objective).

The ROS levels in both urothelium and detrusor smooth muscle were measured using the fluorescent dye DHE in frozen bladder sections (Figure 4A). In the control group, the basal ROS levels were 70% higher in the urothelium than detrusor (*p* < 0.001). Exposure to MGO significantly increased the ROS levels in the urothelium and detrusor smooth muscle compared with the control group, but the increases were higher in detrusor (108%, *p* < 0.001) than urothelium 25% (*p* < 0.05) (Figures 4B,C). The analysis of SOD activity showed a 40% reduction in MGO compared with control group (*p* < 0.01; Figure 4E).

### MGO Exposure Increases mRNA Expression of L-type Ca^2+^ Channels (Cacn1) and Rho-Kinase System in Bladders

**FIGURE 5:**
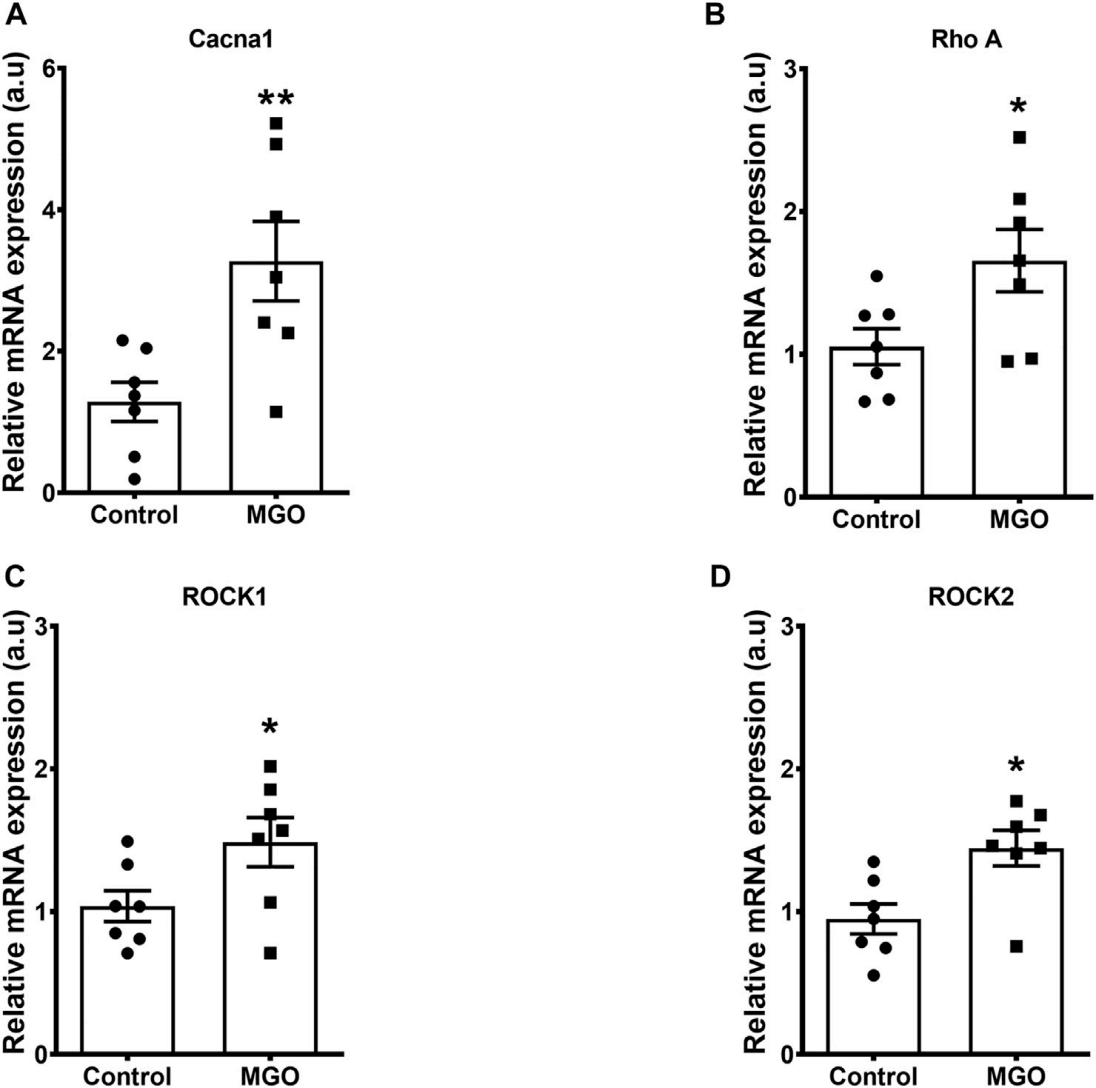
Methylglyoxal (MGO) treatment increases the mRNA expressions of L-type Ca^2+^ channels **(A)**, RhoA **(B)**, ROCK1 **(C)** and ROCK 2 **(D)** in intact bladder tissues. Data are expressed as mean ± SEM for *n* = 7. *p < 0.05 compared with control group.

qPCR analysis of bladders revealed that MGO exposure significantly increased the mRNA expressions of L-type Ca^2+^ channels, RhoA, ROCK1 and ROCK2 when compared to the control group (*p* < 0.05; Figures 5A–D).

### Contractile Responses of Intact and Mucosa-Denuded Bladders

**FIGURE 6:**
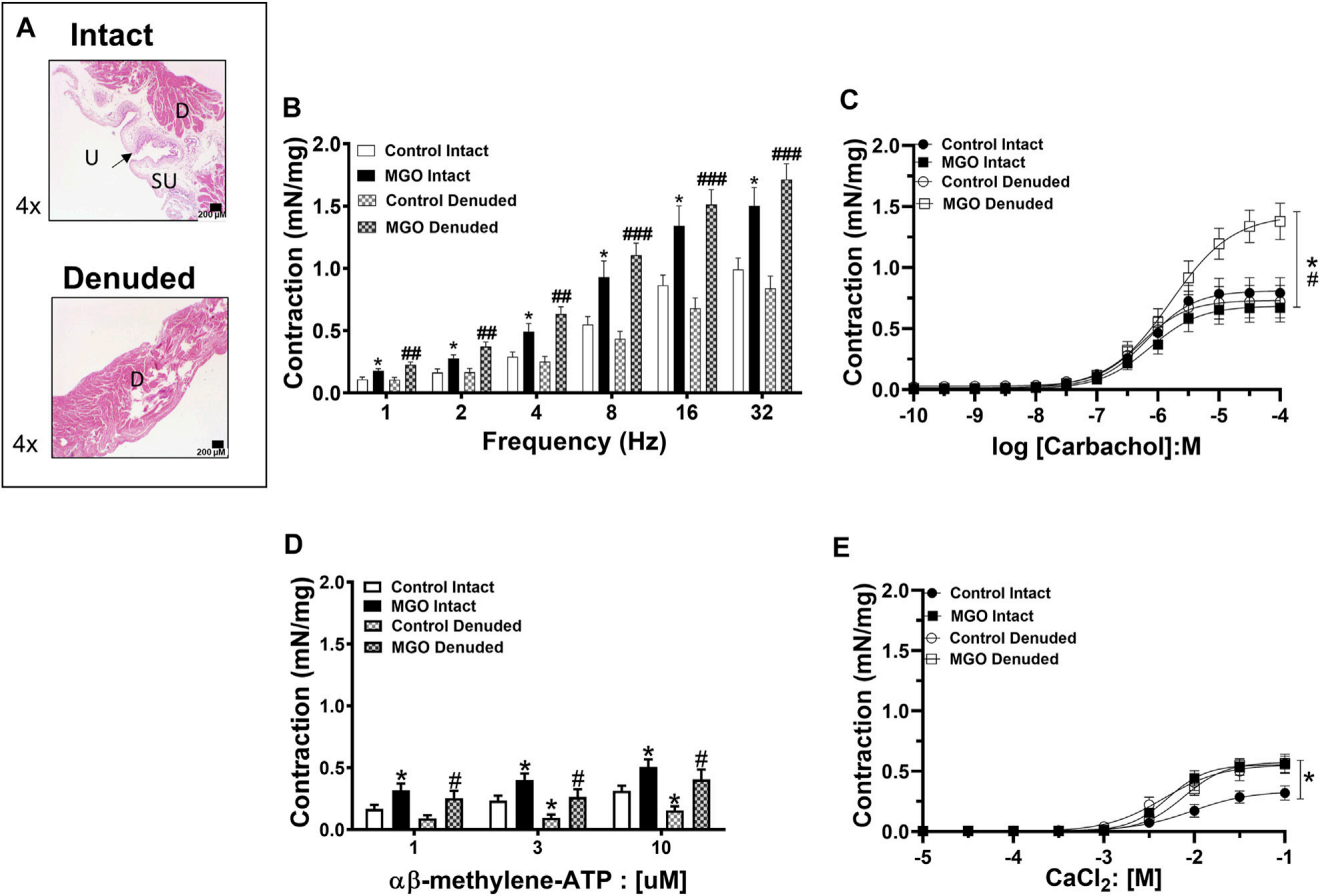
The effects of methylglyoxal (MGO) treatment in intact and mucosa-denuded bladder contractions. Panel **(A)** shows representative images of intact bladders indicating the presence of urothelium, suburothelium and detrusor smooth muscle whereas in denuded bladder only the detrusor can be observed. Panels **(B−E)** show the contractions induced by electrical-field stimulation (EFS; 1–32 Hz), carbachol (0.1–100 µM), α,β-methylene-ATP (1–10 µM) and extracellular Ca^2+^ (10 μM–100 mM), respectively. Data are expressed as mean ± SEM (6–10 mice per group). *p < 0.05 compared with respective control-intact groups; ^#^ *p* < 0.05, ^##^ *p* < 0.05, ^###^ *p* < 0.001 compared with respective control-denuded groups. In panel **A**, urothelium, suburothelium and detrusor smooth muscle are designated as U, SU, and D, respectively. Black bars in panel A represent a scale of 200 μm (×4 objective).

For the functional assays, the bladder mucosa was maintained intact or mechanically removed. Figure 6A shows that mechanical mucosa removal produced no gross damage to the detrusor layer. We then evaluated the contractile responses to electrical-field stimulation (EFS; 1–32 Hz), muscarinic agonist carbachol (0.001–100 μM0, P2X receptor agonist α,β-methylene ATP (1–10 µM), and extracellular Ca^2+^ (0.01–100 mM) in both intact and mucosa-denuded bladder strips from control and MGO-exposed mice.

In the control group, the mucosa removal did not significantly affect the bladder contractile responses to EFS at any frequency. In the MGO group, the contractile responses to EFS were greater than the control group irrespective of whether the mucosa was intact or removed (*p* < 0.05; Figure 6B).

In the control group, the mucosa removal did not significantly affect carbachol-induced bladder contractions. Exposure to MGO did not significantly change the carbachol-induced contractions in mucosa-intact preparations, but it markedly increased the contractions in mucosa-denuded tissues (*p* < 0.01; Figure 6C). The potency value for carbachol was significantly reduced in mucosa-denuded tissues (5.79 ± 0.11; *p* < 0.05) in comparison with the other groups (6.16 ± 0.13, 6.12 ± 0.14 and 6.27 ± 0.15 for control-intact, MGO-intact, and control-denuded strips, respectively).

Exposure to MGO significantly increased the contractile responses to α,β-methylene ATP, as observed in both intact and mucosa-denuded bladder strips (*p* < 0.05; Figure 6D).

In the control group, mucosa removal significantly enhanced the bladder contractions to extracellular Ca^2+^. MGO exposure increased the CaCl_2_-induced contractions in the intact bladder preparations (*p* < 0.05), but it did not further increase the contractions in the mucosa-denuded preparations (Figure 6E).

### Y27632 and PEG-SOD Normalizes the Contractility of Mucosa-Denuded Bladder Strips in MGO

**FIGURE 7:**
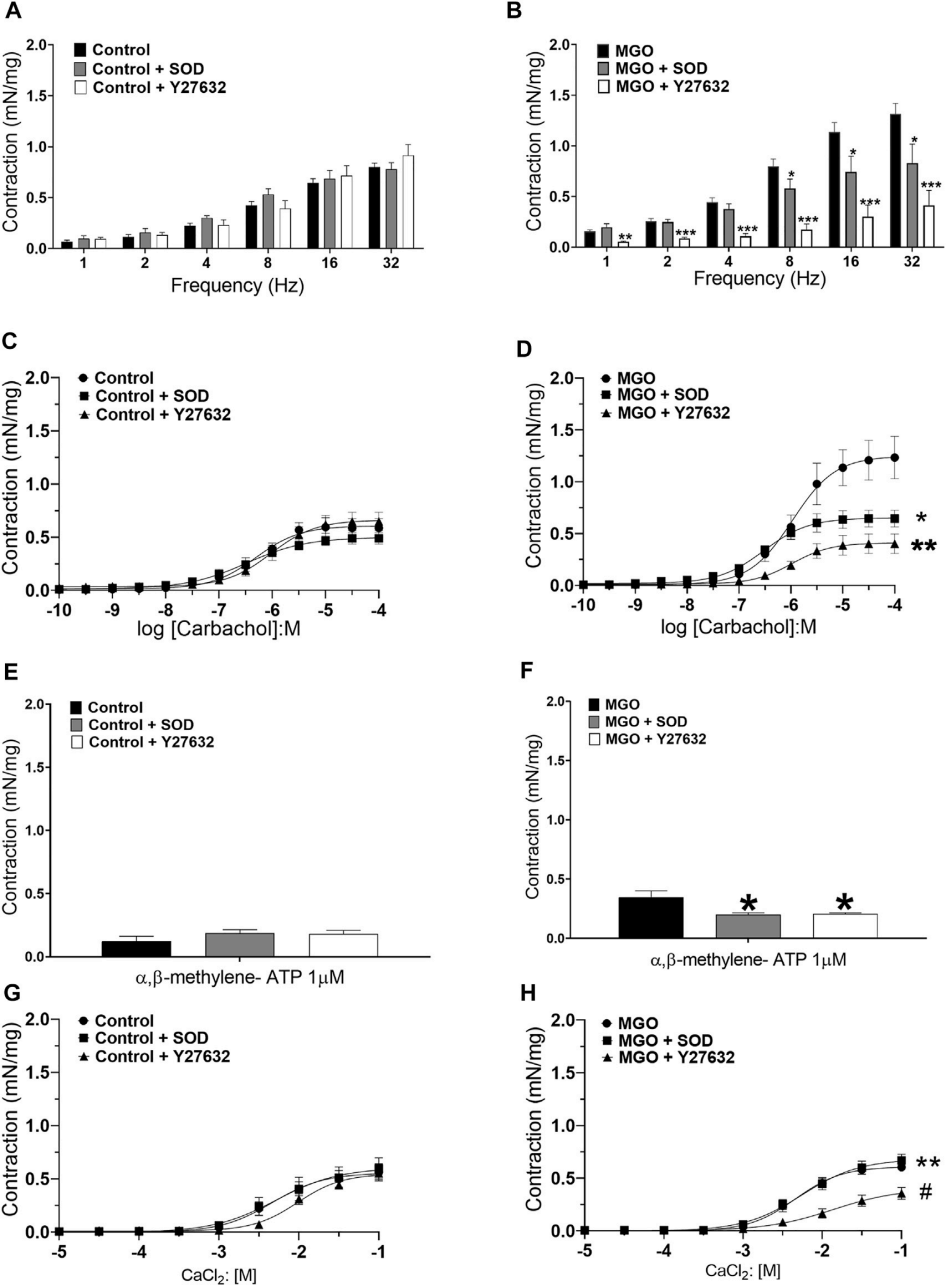
Incubation of mucosa-denuded bladder preparations with superoxide dismutase (PEG-SOD; 87 IU/ml) or the Rho kinase inhibitor Y27632 (1 µM) reduces the enhanced detrusor smooth muscle contractions in mice treated with methylglyoxal (MGO) in comparison with control mice. Panels **(A,C,D and E)** shows the contractions induced by electrical-field stimulation (frequency), carbachol, α,β-methylene-ATP (1–10 µM) and extracellular Ca^2+^ in control mice, respectively. Panels **(B,D,F and H)** shows the contractions induced by the same stimuli in MGO-treated mice. Data are expressed as mean ± SEM (*n* = 5-6 per group). *p < 0.05, **p < 0.01, ***p < 0.001 compared with untreated preparations. ^#^ *p* < 0.05 compared with MGO + SOD group.

The possibility that the increased bladder contractions in MGO-exposed mice reflect enhanced ROS production and Rho kinase activation in the detrusor smooth muscle was investigated in mucosa-denuded preparations preincubated (30 min) with either PEG-SOD (87 IU/ml) or the Rho kinase inhibitor Y27632 (1 µM). In the control group, at the concentrations employed, neither SOD nor Y27632 affected the contractions induced by EFS, carbachol, α,β-methylene ATP, and CaCl_2_ (Figures 7A,C,E,G). However, in the MGO group, preincubation with SOD or Y27632 nearly prevented the bladder contractions to EFS, carbachol, and α,β-methylene ATP (Figures 7B,D,F). In the MGO group, the increased CaCl_2_-induced contraction was not affected by SOD preincubation, but it was normalized by Y27632 (Figure 7H).

## Discussion

The present study demonstrates that oral intake with 0.5% MGO for 12 weeks in female mice increases the serum levels of AGEs and the bladder expressions of RAGE, ROS, RhoA, ROCK1, ROCK2 and L-type Ca^2+^ channels. MGO treatment increased the bladder contraction to EFS, carbachol, and α,β-methylene-ATP, an effect prevented by prior incubation with either SOD or Y27632. Overall, activation of AGE-RAGE-ROS signaling in bladder tissues may be responsible for the resulting MGO-induced detrusor overactivity.

In the present study, we administered 0.5% MGO for 12 weeks in female mice, after which MGO and F-AGEs were measured in serum. Similar to male mice (Oliveira et al., 2021), higher levels of MGO and F-AGEs were found in MGO-exposed mice. No changes in glucose levels (this study) or insulin resistance (Medeiros et al., 2020) were observed in the MGO-treated mice, confirming that elevated serum MGO was indeed generated by the oral intake of MGO rather than by the endogenous glucose metabolism, as observed in diabetes-associated diseases (Yamagishi et al., 2012). Histological analysis of the bladders showed that MGO treatment caused small increases (despite significant) in bladder weight, urothelium thickness, detrusor thickness and collagen content. In bladders of male mice receiving MGO (Oliveira et al., 2021), we detected a similar increase of the urothelium thickness, despite no alteration in the detrusor thickness was observed. With respect to the collagen content, the increases in bladder of males (Oliveira et al., 2021) were markedly higher than the females (this study). We are uncertain about the reasons that explain the histological differences in bladders of male and females, but that may rely on the bioavailability of MGO and formation of AGEs, as well as on the ability of the glyoxalase system to detoxify MGO in each mouse sex.

**FIGURE 8:**
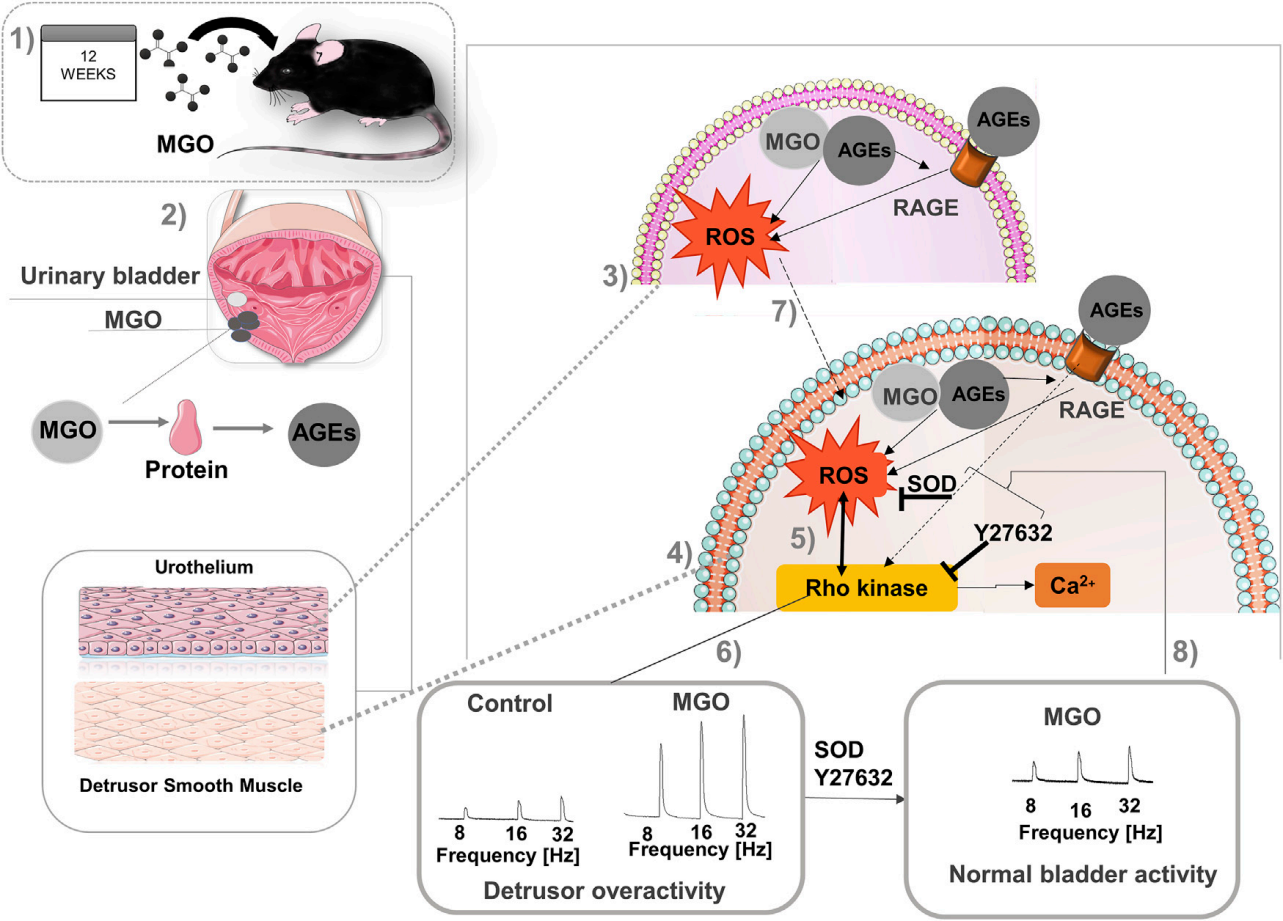
Proposal of mechanisms involved in detrusor overactivity induced prolonged intake of methylglyoxal (MGO) in female mice. Methylglyoxal was given at 0.5% in the drinking water of female mice for 12 weeks (1). In urinary bladder (2), MGO generates advanced glycation end products (AGEs) that interacts with its cell surface receptor, RAGE, in both urothelium (3) and detrusor smooth muscle (4), leading to excess of reactive-oxygen species (ROS) generation in both layers. In detrusor smooth muscle cells, ROS activates the Rho kinase system (5), ultimately leading to Ca^2+^ sensitization and detrusor overactivity (6). Alternatively, RAGE activation may directly activate Rho kinase further elevating the ROS levels. ROS produced in urothelium (7) may also diffuse to detrusor cells amplifying the smooth muscle contraction. Bladder incubation with superoxide dismutase (SOD) or the Rho kinase inhibitor Y27632 significantly reduce the detrusor overactivity, normalizing the bladder activity (8). This image was produced with the assistance of Servier Medical Art (https://smart.servier.com) licensed by a Creative Commons Attribution 3.0 Unported License.

It is well established that accumulation of MGO causes the so-called dicarbonyl stress, which is defined as the abnormal accumulation of dicarbonyl reactive metabolites leading to protein glycation and hence tissue injury (Rabbani and Thornalley, 2015). AGEs bound its cell receptor RAGE in vascular endothelial, initiating tissue injury that may involve increased ROS production (Delbin et al., 2012). Cells have evolved a balanced system to neutralize the extra ROS that consist of enzymatic antioxidants such as SOD, and studies show that antioxidant activity of SOD may be impaired upon ROS excess, thereby dysregulating many physiological processes (He et al., 2017). Except of bladder cancer (Khorramdelazad et al., 2015) and mouse cystitis (Roundy et al., 2013; Hiramoto et al., 2020), no previous study explored the RAGE expression in bladders. Compared to control mice, RAGE immunostaining was higher in both urothelium and detrusor of MGO-treated mice, as well as in the vascular endothelium supplying the detrusor and lamina propria. Analysis of mRNA expressions of RAGE also revealed a higher RAGE expression in bladder tissues of the MGO group. Additionally, using the fluorescent dye DHE in frozen bladder sections, MGO treatment significantly increased the ROS levels in both urothelium and detrusor smooth muscle (despite higher levels were found in the detrusor), which was accompanied by reduced SOD activity in the bladder. Therefore, like vascular tissues, the activation of AGE-RAGE-ROS signaling may be implicated in MGO-induced detrusor overactivity (Figure 8).

Acetylcholine *via* muscarinic M3 receptors is the principal excitatory transmitter at the parasympathetic nerve terminals in detrusor smooth muscle, whereas ATP, *via* P2X1 receptors, mediates the atropine-resistant neurogenic bladder contractions and may be involved in some types of bladder dysfunction (Abrams et al., 2006; Yoshimura et al., 2008). Bladder contractions in response to EFS reflect mainly the release of both acetylcholine and ATP (Leiria et al., 2011). In our study, the bladder contractions induced by EFS were significantly higher in MGO group, as observed both in intact and mucosa-denuded preparations. The contractions induced by the full muscarinic agonist carbachol did not differ between control and MGO groups in intact bladders, but the mucosa removal largely increased the contractions in MGO-treated animals. In addition, the P2X1 agonist α,β-methylene ATP induced higher contractile responses in bladders of MGO group, irrespective if mucosa was removed or not. Of note, our findings that presence of mucosa impairs the bladder contractions by carbachol activation (but not by α,β-methylene ATP) indicates that MGO treatment also affects the urothelium, which under muscarinic receptor activation may releases relaxing factors that counteracts the contractions. Under physiological conditions, the muscarinic and purinergic-mediated bladder smooth muscle contractions depend on extracellular Ca^2+^ influx secondary to L-type Ca^2+^ channel opening (Rapp et al., 2005; Leiria et al., 2011; Yu, 2022). Compared with control animals, in intact bladder preparations of MGO-treated mice we found a higher mRNA expression of L-type Ca^2+^ channels, and that the cumulative addition of extracellular Ca^2+^ in nominally Ca^2+^-free solution produced significantly higher bladder contractions. Interestingly, when the bladder mucosa was removed, MGO failed to increase the contractions above the control levels, suggesting that extracellular Ca^2+^ entry in mucosa favors the release of relaxing mediators that act to reduce the muscle contraction. Urothelium is reported to release inhibitory mediators that modulate the relaxant responses of the bladder (Sellers et al., 2018), including nitric oxide (NO), prostanoids (Guan et al., 2017) and hydrogen peroxide (H_2_S; d'Emmanuele di Villa Bianca et al., 2016), amongst others. Urothelium also expresses β-adrenoceptors, which modulates the relaxant responses to β-adrenoceptor agonists, possibly *via* the release of urothelium-derived factors (Otsuka et al., 2008; Moro et al., 2013). In our study, whether the increased bladder contractions by prolonged intake of MGO is also accompanied by an impairment of urothelium-mediated relaxant responses remain to be investigated.

The Rho kinase pathway is reported to regulate the sensitivity of the Ca^2+^ dependent regulatory system (Wegener et al., 2004). Dysregulation of the Rho kinase system has been implicated in detrusor overactivity in animal models of acetic acid-induced cystitis, stretch-induced bladder contractions, and chronic bladder ischemia (Peters et al., 2006; Shimizu et al., 2013; Shiomi et al., 2013; Wróbel and Rechberger, 2017; Akaihata et al., 2018). Moreover, *in vitro* biochemical assays and *in vitro* studies in vascular smooth muscle have shown an association between increased oxidative stress and upregulation of the RhoA/Rho-kinase system (Heo and Campbell, 2005; Aghajanian et al., 2009; Priviero et al., 2016; MacKay et al., 2017). Accordingly, our findings show higher mRNA expressions of L-type Ca^2+^ channels, RhoA, ROCK1, and ROCK2 in the bladder tissues of the MGO group. We then performed functional assays in mucosa-denuded bladder strips in the absence and in the presence of either SOD or Y27632. At the concentrations employed here, in the control group, both compounds had no effects on EFS-, carbachol- and α,β-methylene ATP-induced contractions. However, in MGO-treated mice, SOD or Y27632 significantly reduced the contractile responses, clearly indicating a major role for superoxide and Rho kinase in the detrusor overactivity (Figure 8). This is consistent with previous studies in pulmonary vascular smooth muscle under chronic hypoxia (MacKay et al., 2017; Yan et al., 2020) and corpus cavernosum of diabetic mice (Li et al., 2012; Priviero et al., 2016) where ROS generation through upregulation of RhoA/ROCK signaling mediated the pulmonary vasoconstriction and impaired cavernosal relaxations. However, in isolated cells, Rho kinase inhibitors like Y27632 can also reduce the ROS production (Lu et al., 2013; Fujimoto et al., 2017). Recently, the ROS-mediated increases of RhoA levels in a murine macrophage cell line were shown to amplify the superoxide production through ROCK phosphorylation of p47phox, maintaining a positive feedback loop for superoxide generation (Cap et al., 2020). Therefore, in our study, additional approaches would be required to elucidate if upregulation of Rho kinase in bladders of MGO-treated mice also amplify the ROS production in positive feedback way. With respect to the contractile responses induced by extracellular Ca^2+^ in mucosa-denuded bladder strips of MGO group, we found that SOD preincubation had no effect, whereas Y27632 rather restored the contractions to control levels. This finding suggests that the resulting bladder contraction due to extracellular Ca^2+^ influx through L-type Ca^2+^ channels is not under the influence of the upstream superoxide anion production in detrusor, but itself can increase the sensitivity to Rho kinase.

In terms of study limitation, MGO here was administered to healthy female mice in the drinking water at 0.5% for 12 weeks, after which plasma and bladder were collected and used for the biochemical, molecular, and functional assays. This model greatly differs from the classical diabetic animal model in that MGO is not generated from the endogenous glucose metabolism but rather exogenously supplied to animals. Despite high serum levels of MGO and AGEs (this study) along with reduced glyoxalase-1 expression and activity (Oliveira et al., 2021) are observed in mice orally taking MGO, which suggest a true dicarbonyl stress (Rabbani and Thornalley, 2015), caution should be taken when comparing our present model with those involving endogenous generations of dicarbonyl species.

## Conclusion

Prolonged MGO intake in mice leads to serum accumulation of MGO and F-AGES, which triggers the activation of RAGE-ROS signaling in the bladder wall, leading to detrusor overactivity, which is normalized by SOD or the Rho kinase inhibitor Y27631 (Figure 8). It is possible that overactive bladder syndrome in diabetes-associated diseases reflects by accumulation of MGO in circulating blood.

## Data Availability

The raw data supporting the conclusions of this article will be made available by the authors, without undue reservation.
